# Characteristics and Whole-Genome Analysis of *Limosilactobacillus fermentum* Phage LFP02

**DOI:** 10.3390/foods12142716

**Published:** 2023-07-16

**Authors:** Ruirui Lv, Xin Gao, Can Zhang, Weiqi Lian, Xingyu Quan, She Guo, Xia Chen

**Affiliations:** 1 Inner Mongolia Key Laboratory of Dairy Biotechnology and Engineering, Inner Mongolia Agricultural University, Hohhot 010018, China; lvruirui0428@163.com (R.L.); gaosanjin666@163.com (X.G.); 17861120569@163.com (C.Z.); 13514868400@163.com (W.L.); qxyqxyjy123@163.com (X.Q.); guoshe0825@163.com (S.G.); 2 Key Laboratory of Dairy Products Processing, Ministry of Agriculture and Rural Affairs, Inner Mongolia Agricultural University, Hohhot 010018, China; 3 Key Laboratory of Dairy Biotechnology and Engineering, Ministry of Education, Inner Mongolia Agricultural University, Hohhot 010018, China

**Keywords:** *Limosilactobacillus fermentum* phage LFP02, infective properties, environmental stress tolerance, adsorption characteristics, whole genome sequence

## Abstract

*Limosilactobacillus fermentum* is a bacterium widely used in food production, medicine, and industrial fermentation. However, fermentation could fail due to phage contamination. *L. fermentum* bacteriophage LFP02 can be induced from *L. fermentum* IMAU 32579 using mitomycin C. To better understand the characteristics of this phage, its physiological and genomic characteristics were evaluated. The results showed that its optimal multiplicity of infection was 0.01, and the burst size was 148.03 ± 2.65 pfu/infective center. Compared to temperature, pH had a more obvious influence on phage viability, although its adsorption capacity was not affected by the divalent cations (Ca^2+^ and Mg^2+^) or chloramphenicol. Its genome size was 43,789 bp and the GC content was 46.06%, including 53 functional proteins. Compared to other *L. fermentum* phages, phage LFP02 had chromosome deletion, insertion, and inversion, which demonstrated that it was a novel phage. This study could expand the knowledge of the biological characteristics of *L. fermentum* bacteriophages and provide some theoretical basis for bacteriophage prevention during fermentation.

## 1. Introduction

*Lactobacillus fermentum* is an obligate heteromorphic Gram-positive bacterium that can ferment galactose, lactose, and other carbohydrates. According to the update of the international classification status of species, *L. fermentum* was renamed *Limosilactobacillus fermentum* [1]. It widely existed in fermented vegetable products, meat products, soybean products, dairy products, human saliva, and the intestines of humans and animals. Previous studies have confirmed that this species is the dominant microorganism in traditional fermented foods [2]. In 2009, the European Food Safety Authority (EFSA) presented it a Qualified Presumption of Safety (QPS) and the US Food and Drug Administration (FDA) admitted that it was a ‘Generally Recognized as Safe’ (GRAS) organism [3].

Phages are a kind of virus, which can infect bacteria and replicate in host cells. As reported, prophage sequences have existed in about 40–50% of microbial genomes [4]. Lysogenic phages have a certain degree of concealability and are difficult to find. Oh et al. predicted the prophage site of 16 *L. reuteri* strains using PHASTER and found 21 presumptive complete prophages [5]. Pei et al. induced 142 LAB stains by MMC and obtained 8 novel phages [6]. Prophages and prophage-like elements have always been found in microbial genomes. Thus, once the prophages in starter cultures were induced into lytic cycles under some stress or specific conditions, they may cause premature cleavage or slow fermentation, and even result in fermentation failure [7,8,9]. Moreover, lysogenic phages can mediate gene transfer between bacteria, which is an event that may increase the virulence of bacteria–for example, by promoting antibiotic resistance [10]. In this study, we used the previous taxonomic status to treat the genus *Lactobacillus* as a whole for subsequent analysis. By 2023, the whole genome sequence of 92 *Lactobacillus* bacteriophages had been published in the NCBI database, yet only 4 whole genomes for *L. fermentum* phages were included.

In our previous study, a complete prophage sequence was observed in the genome of *L. fermentum* IMAU32579 by using PHASTER analysis, after which, we successfully induced phage LFP02 by using MMC (1.9 μg/mL). The aim of this study was to evaluate the biological properties (such as infective properties, tolerance, and adsorption characteristics) and genome characteristics of this phage. In addition, the differences between phage LFP02 and other published *L. fermentum* phages were also analyzed. This study could provide some theoretical basis for improving the stability of fermentation-related products and constructing phage control measures; it also has important guiding significance for the breeding and renewal of anti-bacteriophage strains.

## 2. Materials and Methods

### 2.1. Bacterial Strains, Phages, and Culture Conditions

The host strain *L. fermentum* IMAU 32579 was isolated from traditional fermented milk and was stored in the Lactic Acid Bacteria Collection Center, Key Laboratory of Dairy Biotechnology and Engineering, Ministry of Education, Inner Mongolia Agricultural University, Hohhot, China. Phage LFP02 was induced from *L. fermentum* IMAU 32579. The host strain was cultured in MRS (de Man, Rogosa, and Sharpe) broth at 37 °C for 24 h. Phage LFP02 was added into the bacterial suspension (about OD_600_ ≈ 0.5) and incubated at 37 °C for 3–4 h, then, the bacteriophage lysate was stored at 4 °C. Double agar overlay assay (DLA) was used to evaluate phage titer [11,12].

### 2.2. Bacteriophage Host Range

DLA method was used to evaluate the host range of the phage LFP02 in 52 bacterial strains (Table S1). *L. fermentum* IMAU32579 was used as the positive control. Specifically, 20 μL bacterial culture in the exponential growth phase and 100 μL diluted phage lysate were mixed with semi-solid MRS agar medium and transferred to plates containing a layer of solid MRS agar medium. After solidification, plates were incubated overnight at 37 °C, and then, checked for the presence of lytic plaques.

### 2.3. Determination of Optimal Multiplicity of Infection (MOI)

Host cells were infected by phage particles at different ratios (0.001, 0.01, 0.1, 0.5, 1, 2, 10, and 100). The host bacterial suspension (OD_600_ ≈ 0.5) and phage lysate were diluted in gradient, and then, the bacterial suspension (100 μL) and phage lysate (100 μL) were mixed with MRS liquid medium (800 μL), and incubated at 37 °C for 4 h. Then, the suspensions were centrifuged (8000 rpm for 5 min) and filtered with a sterile filter membrane (0.22 μm), and the supernatant was collected to determine the titer. The MOI resulting in the highest phage titer was considered the optimal MOI [13].

### 2.4. Infective Characteristic

*L. fermentum* IMAU32579 was grown to the exponential stage (OD_600_ ≈ 0.5), then the phage stock solution was added at the optimal MOI. After adsorption at 37 °C for 15 min, cells were collected by centrifugation at 8000 rpm for 5 min. The sediment was resuspended in MRS liquid medium (37 °C). At regular intervals (15 min), 100 μL of each dilution was collected for bacteriophage enumeration. Finally, the latent time, burst time, and burst size were calculated by the methods previously described by Lu et al. [13].

### 2.5. Environment Stress Tolerance

In order to evaluate the effect of temperature and pH values on phage viability, this study used the methods previously described by Lv et al. [14]. Viable phages were counted immediately using the DLA method.

### 2.6. Factors Influencing Phage Adsorption

*L. fermentum* culture (under optimal MOI) was suspended in MRS liquid medium (30 min) to evaluate the effects of temperature and pH on phage adsorption. The adsorption of phage LFP02 was evaluated at different temperatures (0, 10, 20, 30, 37, 42, and 50 °C) and different pH values (4–11).

In order to explore the effect of divalent cations (Ca^2+^ and Mg^2+^) and chloramphenicol on the adsorption of phage, this study used the methods previously described by Lv et al. [14]. The minimum chloramphenicol concentration was determined based on previous studies [15].

For all the evaluated factors, adsorption rates were evaluated according to the methods described by Capra et al. [16] Finally, the changes in phage particles with time under different conditions (temperature, pH, divalent cations, and chloramphenicol) were calculated.

### 2.7. Genome Sequencing and Assembly

In this study, the saturated phenol–chloroform extraction method was used to extract phage DNA [17], and the phage genome was sequenced by PacBio SMRT (Pacific Biosciences, Menlo Park, CA, USA) platform in Anshan Biotechnology Co., Ltd. (Tianjin, China). Finally, the phage genome was assembled using Flye 2.8.3 [18].

### 2.8. Bioinformatic Analysis

Identification of functional proteins was carried out using Nr Database (Non-Redundant Protein Database) (https://www.ncbi.nlm.nih.gov/protein, accessed on 3 September 2021) and RAST (http://rast.nmpdr.org/, accessed on 3 September 2021) [19]. Using CGView Server (http://cgview.ca, accessed on 23 September 2022 ) to construct a genome map and visualize it. The Synteny Block was implemented by MAUVE (https://asap.genetics.wisc.edu/, accessed on 14 December 2022) [20].

### 2.9. Statistical Analysis

The data were analyzed by SPSS 20.0 software (one-way analysis of variance, ANOVA), and the graphs were drawn by using Origin 9.0. All experiments were performed in triplicate.

## 3. Results and Discussion

### 3.1. Phage Host Range

The host range can provide insights into the relationship between the origins of new phages with host strains [21]. In this study, 52 *Lactobacillus* sp. strains were used for the host range test (Table S1). The results showed that phage LFP02 only infected *L. fermentum* IMAU32616, *L. fermentum* IMAU32157, and *L. fermentum* IMAU32649. Capra et al. reported that *L. paracasei* phage MCL-A could infect 7 strains of *L. paracasei* (16 strains were evaluated) and 2 strains of *L. casei* (6 strains were evaluated) [22]. Compared to previous studies, the host range of phage LFP02 was narrow, and it could not infect other bacterial species. All the strains that it infected were isolated from Xinjiang traditional fermented milk, which is often eaten by herdsmen and whose fermentation environment is more open than in factories. Sample type, fermentation conditions, sanitary environment, and residual initial culture are all involved in the development of microorganisms in fermented milk [23]. Therefore, the microbial community structure of fermented milk from nearby areas was usually more similar. As the results show, phage LFP02 only infected three *L. fermentum* strains. This phenomenon may be due to the internal defense mechanisms of some bacteria, e.g., restrictive modification systems, CRISPR/Cas, or resident prophage, which may endow the strains with phage resistance [24].

### 3.2. Determination of Optimal Multiplicity of Infection (MOI)

**Figure 1:**
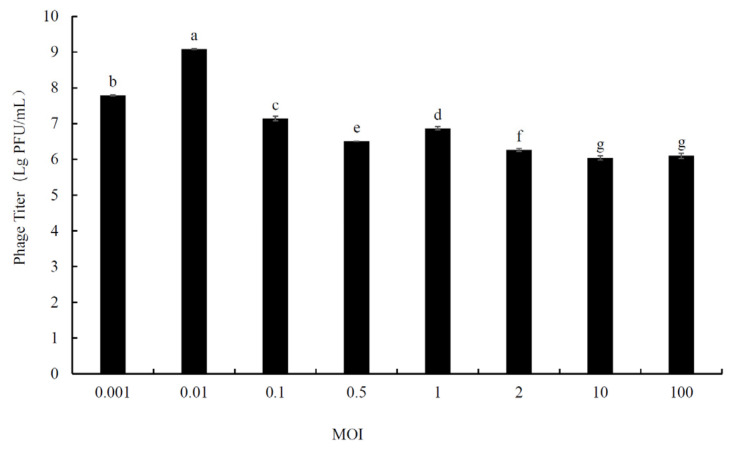
The multiplicity of infection (MOI) of *L. fermentum* phage LFP02. Note: the significant differences (*p* < 0.05) among the groups are expressed by different lowercase letters.

MOI is defined as the ratio of phage particles to host cells [25]. The results showed that the optimal MOI of *L. fermentum* phage LFP02 was 0.01, and the phage titer was 1.22 × 10^9^ pfu/mL (Figure 1). Similar to our results, Foschino et al. reported the optimal MOI for *L. sanfranciscensis* phage EV3, which was isolated from sourdough, was 0.01 [26]. Liu et al. found that the optimal MOI of phage phiTY18 was 0.01 [27]. The optimal MOI for *L. brevis* phage was 10 [28]. The MOI is different for different phages, whereby a larger MOI indicates a weaker infection ability.

At the same time, Figure 1 showed that with the increase in MOI, the titer of the LFP02 phage had two peaks, yet the maximum titer was only one. A similar situation also appeared in the studies by Liu et al. [27] and Mercanti et al. [29]. As for the reasons for the decline, Mercanti et al. suggested that this may be due to the limited number of attachment sites available to phages on the cell wall, or another type of spatial obstruction [29].

### 3.3. Infective Characteristic

The results showed that the latent period of phage LFP02 was 30 min, the burst time was 60 min (from 30 min to 90 min), and the burst size was 148.03 ± 2.65 pfu/infective center. The transition of the phage from adsorption to release is an explosion process, and its duration and degree will be directly related to the subsequent effect of the phages. At present, there is a lack of research on the dynamics of phage lysis in *L. fermentum*. In 2010, De Antoni found *L. plantarum* phage FAGK1 and FAGK2 both had 30 min latent periods and burst times between 30 and 110 min. Their burst sizes were 10.8 pfu/infective center and 12 pfu/infective center, respectively [30]. In 2019, Sunthornthummas reported a burst size of the *L. paracasei* phage φT25 of 38 pfu/infective center [31]. These previous examples suggest that phage LFP02 has a shorter latent and lysis period, yet that the amount of lysis was greater. Once this phage contaminates the fermentation, it may cause huge economic losses.

### 3.4. Environmental Stress Tolerance

Environmental factors could affect a phage’s viability. However, there are few reports on these data for *L. fermentum* phages. Therefore, it is very important to explore the effects of environmental factors on the survival rate of phage LFP02.

#### 3.4.1. Thermal Stability

**Figure 2:**
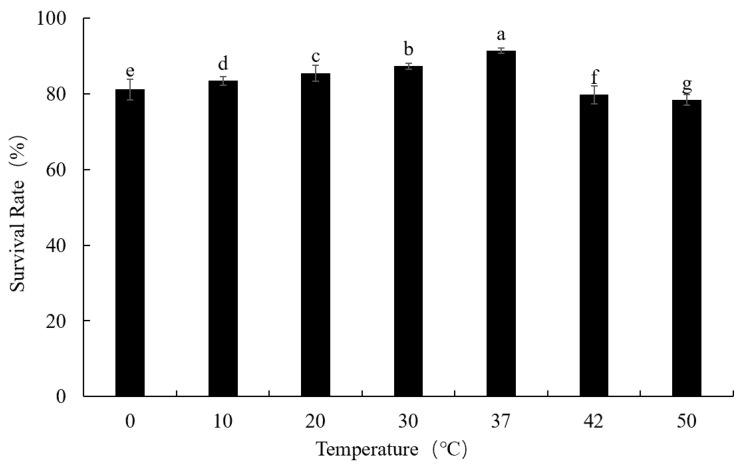
Thermal stability of phage LFP02. Note: the significant differences (*p* < 0.05) among the groups are expressed by different lowercase letters.

**Figure 3:**
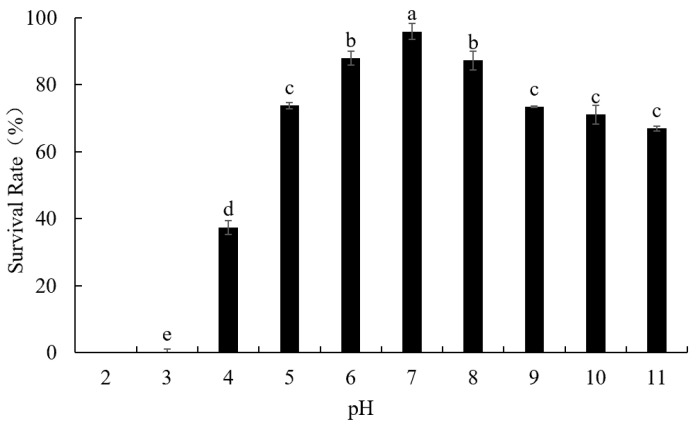
pH stability of phage LFP02. Note: the significant differences (*p* < 0.05) among the groups are expressed by different lowercase letters.

Figure 2 showed that the survival rate of phage LFP02 was 91.34% at 37 °C (Figure 3). When the temperature was increased to 50 °C, the survival rate was reduced to 78.36%. The survival rate of this phage was significantly affected by temperature (*p* < 0.05). Müller-Merbach et al. found that *Lactococcus lactis* phage P008 reduced only 1 log_10_ at 55 °C for 3 h [32]. Briggiler Marcó et al. also found that the survival of *L. plantarum* bacteriophages (B1, B2, FAGK1, and FAGK2) was 95% after incubation at 50 °C for 30 min [14]. Therefore, compared to other *Lactobacillus* phages, phage LFP02 is more sensitive to temperature. Previous studies reported that high temperatures may affect the phage by altering its structure and function [33]. Thus, the results obtained in this study may infer that temperature changes affected the structure of the related proteins (such as binding protein, phage tail protein, and phage capsid protein), which decreased the viability of phage LFP02.

#### 3.4.2. Phage LFP02 Stability at Different pH Values

Environmental pH is considered an important factor that affects the stability of phages. In this study, phage LFP02 was held at different pH values (at 37 °C for 30 min), and the survival rate was highest at pH 7 (95.9%) (Figure 3). When pH was reduced to 3, the survival rate was only 0.06%. At pH 2, the phage was completely inactivated. When the pH value increased to 11, the survival rate was 66.97%. In general, phage LFP02 was significantly affected by pH value (*p* < 0.05) and is more resistant to alkaline environments than acid conditions. Previous studies have shown that pH values from 3 to 11 had little effect on *L. paracasei* bacteriophage φT25 but could completely inactivate this phage at pH 2 [34]. Similar to our results, Ruan et al. found *Bacillus cereus* phage SWEP1 was stable in the range of pH 5–10 but was significantly destroyed when the pH value decreased to 4 [35]. In 2021, Fernández reported that the isoelectric point of phage capsid proteins was pH 5.24 [36]. Thus, when the pH drops below this value, the protein changes from a negative to a net positive charge. Moreover, H^+^ concentration can also promote precipitation or aggregation of virus virions, leading to phage inactivation [36,37]. Schmitz et al. reported that acid could destroy the susceptible amino acids, cysteine and methionine, on the phage capsid, thereby causing structural changes, and finally, resulting in its inactivation [38]. In 1970, Laemmli found acidic conditions could cause protein denaturation of the phage capsid, meaning they were more stable under alkaline or neutral conditions [39]. This also suggests that acidic reagents may be more effective in inactivating *Lactobacillus* phages during factory disinfection.

### 3.5. Factors Influencing Phage Adsorption

Phage adsorption is the “first encounter” between the host and the phage, and it is the key step in the phage infecting the host. Adsorption is generally divided into initial contact, reversible adsorption, and irreversible adsorption [40]. In this process, many environmental factors can affect the adsorption process but different bacteriophages have different responses to these factors.

#### 3.5.1. Influence of Temperature on Phage Adsorption

Table 1 shows the adsorption rate of phage LFP02 was maintained at 94% or higher in all the tested temperatures. At 37 °C, this value achieved the highest level (99.1%). However, as the temperature increased, the survival rate decreased. At 50 °C, the lowest value was obtained (94%). Previous research reported that temperature had little effect on *L. paracasei* phage ΦiLp1308 between 0 and 45 °C, yet temperatures above 45 °C expressed a negative effect on its adsorption [29]. Between 10 and 37 °C, the adsorption rate of *L. casei* bacteriophage Lcb ranged from 70 to 83.51%. At 30 °C, the highest adsorption rate (83.51%) was achieved [41].

As is known, phages are composed of proteins and nucleic acids. The temperature might change the structural components of phage, which can influence their infective abilities. Different temperatures will affect the structure of receptor-binding proteins on the surface of phages, thus, affecting their adsorption rates. Tomat et al. suggested that the reduction in phage adsorption rate at 50 °C may be due to disorganization and/or partial degeneration of the phage receptors on the surface of bacterial cells, thus, hindering phage adsorption [42]. Vörös et al. concluded that high temperatures could destruct the phage capsid (such as phage tail protein) and inhibit its infective ability [43]. In addition, temperature affects the adsorption ability of phages, which may be related to the number of receptors on the surface of the host cells and their physiological state. As reported, temperature could influence the random movement of phage particles, and alter the binding and release events between phage-binding proteins and host cell receptors [42,44].

#### 3.5.2. Influence of pH on Phage Adsorption

As shown in Table 2, the adsorption rate of phage LFP02 reached the highest value (99.3%) at pH 7. The adsorption rate decreased slightly with the increase in pH value. Even at pH = 11, the adsorption rate was still 95.1%. Therefore, we can conclude that pH values had little effect on the adsorption rate of this phage.

Trucco et al. found that the maximum adsorption rates of phages CB1/204 and Cb1342 were achieved at pH 4–7, and when the pH increased to 8, the adsorption rates of both phages decreased [45]. The adsorption of *L. paracasei* phages ΦiLp84 and ΦiLp1308 was strongly inhibited at pH 4 and relatively stable at pH 5–8 [29]. Acidic or alkaline agents would inhibit phage adsorption. However, compared to other phages, pH changes had little effect on the adsorption rate of LFP02. Phage adsorption to host bacteria is accomplished by non-specific electrostatic adsorption, which enables the receptor and ligand to bind specifically. Different pH values may cause changes in the adsorption sites or receptor charges on the surfaces of the host and phage, thereby affecting the electrostatic interaction between them.

#### 3.5.3. Influence of Divalent Cations on Phage Adsorption

Since cell receptor and phage adsorption proteins of host bacteria tend to be negatively charged, the addition of divalent cations may increase phage adsorption to the cell surface by electrostatic attraction. From Table 3, we can find that the adsorption rate of the control group was 99.6%, which was higher than from adding Ca^2+^ or Mg^2+^ (*p* < 0.05), indicating that divalent cations did not significantly improve the adsorption capacity of phage LFP02. Similar to our results, Quiberoni reported that Ca^2+^ had no effect on the adsorption kinetics of phage BYM and phage LL-H [41]. Briggiler Marcó et al. found that regardless of whether calcium ions were added, the adsorption rate of phages would reach 99% at 15 min [14]. In 2016, Chen et al. reported that divalent cations were not necessary for the adsorption of *L. plantarum* phage P1 but could accelerate the lytic process [46]. Zhang et al. found that for *L. casei* phage Lcb, Ca^2+^, and Mg^2+^ could promote cell lysis and plaque formation, yet were not necessary for complete lysis [47]. While *Pseudoalteromonas* phage PM2 relied on Ca^2+^ to complete the phage infection process, Mg^2+^ accelerated the adsorption rate of phage DRL-P1 [48,49]. These results indicate that the demand for Ca^2+^ and Mg^2+^ was phage-specific. Although Ca^2+^ and other divalent cations have always been associated with successful phage infection of bacterial cells, certain phages, such as phages of *Escherichia coli*, *Bacillus subtilis*, and *Lactobacillus* spp., can adsorb in the absence of divalent cations [50]. Members of the *Lactococcus* 936 phage group (isolated from industrially produced fermented milk) revealed that Ca^2+^ was required for efficient production of phage progeny but was not necessary for adsorption [51]. Moreover, most *Lactobacillus* phages did not need Ca^2+^ to form plaques, although it was conducive to phage development in the host [52]. During adsorption, the cationic requirements of phages vary depending on the bacterial surface [42]. This flexibility enables phages to survive in a calcium-limited environment while sustaining phages as the most abundant biological organisms on Earth.

#### 3.5.4. Influence of Cell Protein Synthesis on Phage Adsorption

Chloramphenicol expressed no significant effect on the adsorption capacity of phage LFP02 (*p* > 0.05). When the chloramphenicol concentration was 20 µg/mL and 100 µg/mL, phage adsorption rates were still 98.7% and 98.6%, respectively. Similar to our results, Tomat et al. found that chloramphenicol had no effect on the phage adsorption process [42]. Thus, we can conclude that protein synthesis was not necessary for the adsorption process.

### 3.6. Genome Analysis

#### 3.6.1. Genome Organization of Phage LFP02

**Figure 4:**
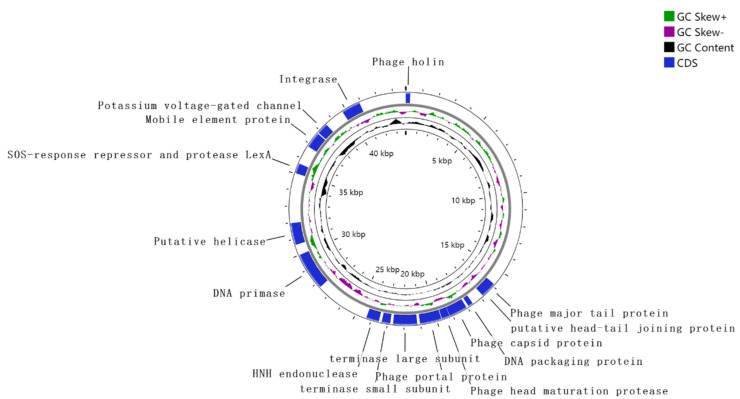
Complete genome circle of phage LFP02.

As shown in Figure 4, phage LFP02 comprised double-stranded DNA (dsDNA), and its genome size was 43,789 bp, with a G + C content of 46.06%. Moreover, 53 functional proteins were annotated (Table S2). Phage LFP02 has a typical genomic structure, including the following modules: packaging, structural protein, lysis, integration, and replication/modification/regulation. Previous reports have highlighted that the small terminase subunit (gene 21), large terminase subunit (gene 22), and HNH endonuclease (gene 23) represent the phage DNA packaging module. The phage terminase subunit consists of two proteins: the large terminase subunit that has the endonuclease domain and ATPase, which provides the power for the DNA packaging reaction; the small terminase subunit, which mediates the specific DNA binding required to recognize the packaging sites in the phage genome [53,54]. These are key enzymes that activate DNA packaging. Similarly, HNH endonuclease (gene 23) is a key component of the phage DNA packaging machinery [55]. Capsid protein (gene 18), head maturation protease (gene 19), and portal protein (gene 20) are associated with the formation of the phage head [56]. Portal protein plays a central role in DNA packaging and capsid formation. In addition, the phage LFP02 genome encoded the HK97 gp10 family phage protein (gene 15), which increased capsid stability and is related to morphology, thereby enabling more efficient and accurate capsid assembly [57,58]. DNA packaging protein (gene 17) and the putative head–tail joining protein (gene 14) form the neck region. Moreover, gene 1 encoded holin, a small transmembrane protein, which participates in the last stage of the phage lysis cycle dsDNA, leading to the lysis of bacterial membranes and the release of the phage progeny into the extracellular [59]. In the integration process of phage LFP02, the site-specific recombination between phage integration sites and bacterial genome integration sites was catalyzed by coding integrase (gene 49 and gene 50) [54]. Phage LFP02 was a lysogenic phage induced by MMC, and its SOS-response repressor and protease LexA were annotated. A previous report showed that lysogen treated with MMC triggered LexA cleavage as part of the host’s SOS response, which induced phage GIL01 to replicate and eventually lyse cells [60,61].

It is worth noting that gene 14, gene 24, and gene 27 are potentially derived from *L. oris*, which was found in the genome of phage LFP02. *L. oris*, which was commonly present in milk, was a potential probiotic strain [62]. Phage LFP02 encoded gene 39, which is related to *L. equigeneros* and is a potential strain for developing equine probiotics [63]. This phenomenon occurred as a result of the open fermentation environment (pastoral area) of traditional fermented milk. Other *Lactobacillus* sp. were mixed into fermented milk through air transmission, and further integrated into the genome of phage LFP02 during the fermentation. The prokaryotic insertion sequence (IS) was one of the smallest transposition elements, which could lead to gene activation, inhibition, or deletion in bacteria [64]. The transposase (IS30 family), derived from *L. plantarum*, was observed in the phage LFP02 genome and contributes to genetic variation in bacteria. Similar to this study, Briggiler Marcó et al. found that *Pediococcus* and *L. plantarum* often exist in the same ecological niche (cucumber fermentation and silage starter culture) and there was an exchange of genetic elements in the genome by phages [65]. Overall, temperate phages were also considered as gene banks for horizontal gene transfer and to have played an important role in horizontal gene transfer (HGT) [24,66]. Phages also represent an important mechanism for the transmission of resistance genes and virulence factors; however, resistant genes and virulent genes were not found in phage LFP02.

#### 3.6.2. Genomic Differences amongst *L. fermentum* Phages

Currently, four *L. fermentum* bacteriophages, all from the *Siphoviridae* family, have been published in the NCBI database: named phages JNU_P1 and JNU_P5 (from human feces) [6], phage LF1 (from Korean kimchi) [67], and phage phiPYB5 (from yogurt) [68].

**Figure 5:**
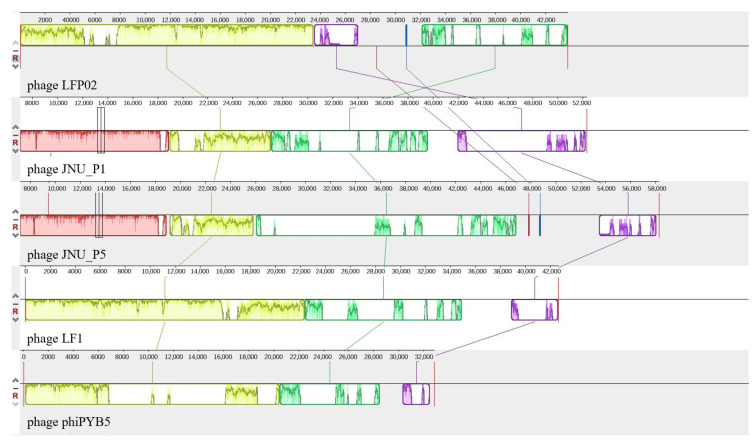
Synteny block of *L. fermentum* phages.

To further expand our genome-level understanding of *L. fermentum* phages, the average nucleotide identity (ANI) of these five phages was calculated (Table S3). We found that the ANI values of phage LFP02 with phage LF1, phage phiPYB5, phage JNU_P1, and phage JNU_P5 were 88.53%, 89.10%, 81.45%, and 87.98%, respectively. Using phage LFP02 as a reference, the synteny block of the genomes of the four bacteriophages from *L. fermentum* showed that other bacteriophages had recombination phenomena, such as chromosome inversion, insertion, and fragment deletion (Figure 5). The genome of phage LFP02 contained about 9500 bp of the specific gene fragment. The main reason for this phenomenon may be because the host bacteria of each phage live in different environments, which prevents gene flow between them [69]. Of these, the host of phages JNU_P1 and JNU_P5 were both isolated from *L. fermentum* in the human intestine, these phages were most different from the other phages and contained a particular fragment, which was not detected in the other three phages. An earlier study that compared the genomes of 54 *Actinomycetes* phages found that their nucleotide sequences had no obvious similarity, suggesting at least a temporary genetic isolation between phages from different hosts [70]. This is consistent with the results of this study where the genomes of the five phages from different sources had differences due to environmental isolation. Interestingly, the genome of phage LFP02 contained three genes encoding proteins (head-tail joining protein, terminase large subunit, and hypothetical protein) that were also found in phage LF1, and one gene encoding protein (capsid protein) that was also found in phage phiPYB5. Correspondingly, the ANI values of phages LFP02, LF1, and phiPYB5 were higher than those for JNU_P1 and JNU_P5 (Table S3). Phage LF1 and phiPYB5 were isolated from Korean kimchi and yogurt, respectively. In this study, the phage hosts were isolated from fermented milk, which may mean that the fermented food environment can lead to more similar *L. fermentum* genomes, resulting in lysogenic phage genomes and functional proteins that were also more similar. Dion et al. believed that there was no sequence homology between different phage morphologies, and some viral proteins were highly conserved at the structural level [71]. These results indicated that phage LFP02 is a novel phage.

## 4. Conclusions

This study focused on the biological and genomic characteristics of *L. fermentum* phage LFP02. Compared to temperature, pH had a more obvious influence on phage viability. It was stable under pH 5–8, although completely lost its activity at pH 2. Moreover, environmental factors (temperature, pH, divalent cations (Ca^2+^, Mg^2+^), and chloramphenicol) expressed little effect on its adsorption. Compared to other *L. fermentum* phages, the genome of phage LFP02 had undergone inversion, insertion, and fragment deletion. Therefore, this phage was a novel lysogenic phage. This study could increase our knowledge about the biological characteristics and genomic diversity of *L. fermentum* phages and provide some theoretical foundation for future research on phage control and screening of phage-resistant strains.

## Figures and Tables

**Table 1:** Effects of temperature on the adsorption rate of phage LFP02.

Temperature (°C)	10	20	30	37	42	50
Adsorption rate	98.39% ± 1.30 ^b^	98.87% ± 1.70 ^a^	98.86% ± 2.00 ^a^	99.03% ± 0.50 ^a^	98.96% ± 1.30 ^a^	94.01% ± 1.12 ^c^

Note: the significant differences (*p* < 0.05) among the groups are expressed by different lowercase letters.

**Table 2:** Effects of pH on the adsorption rate of phage LFP02.

pH	4	5	6	7	8	9	10	11
Adsorption rate	97.00% ± 1.90 ^a^	97.10% ± 1.50 ^a^	98.60% ± 1.12 ^a^	99.30% ± 1.30 ^a^	99.00% ± 1.70 ^a^	98.40% ± 2.00 ^a^	95.90% ± 2.50 ^a^	95.10% ± 1.30 ^a^

Note: the significant differences (*p* < 0.05) among the groups are expressed by different lowercase letters.

**Table 3:** Effects of divalent cations on the adsorption rate of phage LFP02.

Group	Adsorption Rate		
	0 min	15 min	30 min
Control	0%	98.90% ± 0.12 ^a^	99.60% ± 0.03 ^a^
Ca^2+^	0%	97.90% ± 0.09 ^b^	99.00% ± 0.06 ^b^
Mg^2+^	0%	97.00% ± 0.32 ^c^	99.10% ± 0.08 ^b^

Note: the significant differences (*p* < 0.05) among the groups are expressed by different lowercase letters.

## Data Availability

On 10 December 2021, the genome sequence of phage LFP02 was submitted to the Genbank database (https://www.ncbi.nlm.nih.gov/, accessed on 10 December 2021 ) with the accession number SAMN23894542.

## Supplementary Materials

The following supporting information can be downloaded at: https://www.mdpi.com/article/10.3390/foods12142716/s1, Table S1: Strains used in the host range study; Table S2: Phage LFP02 Annotation results; Table S3: Average nucleotide identity of five phages.
